# MOTS-c regulates pancreatic alpha and beta cell functions in vitro

**DOI:** 10.1007/s00418-024-02274-0

**Published:** 2024-03-02

**Authors:** Jakub Bień, Ewa Pruszyńska-Oszmałek, Paweł Kołodziejski, Natalia Leciejewska, Dawid Szczepankiewicz, Maciej Sassek

**Affiliations:** Department of Animal Physiology, Biochemistry and Biostructure, Poznan University of Life Sciences, Poznan, Poland

**Keywords:** MOTS-c, Pancreas, INS-1E, αTC-1

## Abstract

The aim of this study is to determine the influence of the mitochondrial open-reading-frame of the twelve S rRNA-c (MOTS-c) peptide on pancreatic cell physiology. Moreover, in this study, we examined the changes in MOTS-c secretion and expression under different conditions. Our experiments were conducted using laboratory cell line cultures, specifically the INS-1E and αTC-1 cell lines, which represent β and α pancreatic cells, respectively. As the pancreas is an endocrine organ, we also tested its hormone regulation capabilities. Furthermore, we assessed the secretion of MOTS-c after incubating the cells with glucose and free fatty acids. Additionally, we examined key cell culture parameters such as cell viability, proliferation, and apoptosis. The results obtained from this study show that MOTS-c has a significant impact on the physiology of pancreatic cells. Specifically, it lowers insulin secretion and expression in INS-1E cells and enhances glucagon secretion and expression in αTC-1 cells. Furthermore, MOTS-c affects cell viability and apoptosis. Interestingly, insulin and glucagon affect the MOTS-c secretion as well as glucose and free fatty acids. These experiments clearly show that MOTS-c is an important regulator of pancreatic metabolism, and there are numerous properties of MOTS-c yet to be discovered.

**Supplementary Information:**

The online version contains supplementary material available at 10.1007/s00418-024-02274-0.

## Introduction

The human mitochondrial genome is an incredibly fascinating subject of research. Despite being sequenced 40 years ago, it continues to yield discoveries. It consists of 16,569 bp (Anderson et al. 1981) and is known to encode 22 tRNAs, 2 rRNAs, and 13 proteins (Cobb et al. 2016). Delving deeper into the sequences of these 37 genes, researchers have identified smaller functional genes that encode peptides. Among the first to be discovered was the humanin gene (HN), which was identified in Nishimoto’s lab in 2001 through functional expression screening (Hashimoto et al. 2001). Humanin has been confirmed as a biologically active peptide that, for example, enhances insulin sensitivity (Muzumdar et al. 2009).

In 2016, another significant finding occurred with the discovery of six new peptides in the 16S rRNA. These were named small humanin-like peptides (SHLPs). Notably, one of these peptides, SHLP-2, also acts as an insulin sensitizer (Cobb et al. 2016). Another mitochondrial-derived peptide (MDP) is mitochondrial open-reading-frame of the twelve S rRNA-c (MOTS-c). This peptide was discovered through bioinformatic methods in silico in 2015 by Lee et al. (2015). Their research revealed a short open reading frame (sORF) in the human 12S rRNA, consisting of 51 bp, which translates into a peptide comprising 16 amino acids. Interestingly, the translation does not occur using the mitochondria-specific genetic code but it is effective in the cytoplasm using the standard genetic code, making MOTS-c viable.

One of the remarkable aspects of MOTS-c is its highly conserved amino acid sequence, especially in the first 11 positions, across different animal species such as humans, bonobos, orangutans, mice, and rats (Lee et al. 2015).

Although the discovery of MOTS-c occurred just a few years ago, it has already been established as a biologically active peptide. Since mitochondria are present in almost all eukaryotic cells, mitochondria genome and MOTS-c is probably a very important regulator of metabolism. It is expressed in various tissues of laboratory rodents and can be detected in the blood plasma of both humans and rats (Lee et al. 2015; Kim et al. 2019).

Notably, the concentration of MOTS-c in human plasma is found to be decreased in obese people, as observed in both children and adolescent study groups (Du et al. 2018; Luo et al. 2023). Furthermore, in mice, MOTS-c has been shown to counteract diet-induced obesity and insulin resistance (Lee et al. 2015; Yang et al. 2021). Another interesting finding is that, in a mouse model, MOTS-c prevents obesity and insulin resistance induced by ovariectomy (Lu et al. 2019b). In both cases, the action of MOTS-c is mediated through the activation of 5′ adenosine monophosphate-activated protein kinase (AMPK) (Lee et al. 2015; 2019b).

These reports on the effects of MOTS-c on metabolism, especially insulin resistance and energy homeostasis, sparked our interest in investigating the impact of MOTS-c on a significant organ such as the pancreas. The pancreas is a very specific organ with a dual function, serving as both an endocrine and an exocrine gland. In our work, we specifically focused on the endocrine part, particularly on pancreatic islets, and especially on beta and alpha cells. These cells play a vital role in glucose metabolism as they are responsible for the secretion of insulin and glucagon.

The impact of MOTS-c on the pancreas and its secretory functions has not been investigated so far. To address this gap, we performed experiments on pancreatic cell lines, namely INS-1E and αTC-1, which serve as reliable models for studying pancreatic β and α cells, respectively.

## Materials and methods

### Cell culture

The cell lines were cultured in an incubator set at 37 °C with either 5% or 10% CO_2_. INS-1E and αTC1 clone 9 cells were purchased from Sigma Aldrich (Saint Louis, MO, USA) and ATCC (Manassas, VA, USA), respectively. Unless otherwise mentioned, all medium components were purchased from Sigma-Aldrich (Saint Louis, MO, USA).

The INS-1E (Sigma Aldrich, SCC491) cells were cultured using Roswell Park Memorial Institute (RPMI) 1640 medium (Corning, New York, USA), supplemented with 2 g/l NaHCO_3_, glutamine, 1% antibiotic–antimycotic, 1% sodium pyruvate, 1% 4-(2-hydroxyethyl)-1-piperazineethanesulfonic acid (HEPES), and 0.1% β-mercaptoethanol, along with 10% fetal calf serum. The culture medium was replaced twice a week. For experimental purposes, the medium’s composition remained the same as the culture medium, except that 10% fetal calf serum was replaced with 0.2% bovine serum albumin, which was free from free fatty acids. For experiments with variable glucose, RPMI 1640 medium without glucose was used and glucose was supplemented to 2 mM, 6 mM, and 16 mM values.

αTC1 cells clone 9 (ATCC, CRL-2350) were cultured using Dulbecco’s modified Eagle’s medium (DMEM) low glucose medium (Corning, NY, USA). The medium was supplemented with 15 mM HEPES, 0.1 mM nonessential amino acids, 10% fetal calf serum, 0.02% bovine serum albumin that was free from free fatty acids, and 1% antibiotic–antimycotic. The culture medium was replaced twice a week. For the experimental conditions, the medium’s composition remained the same as the culture medium but without the addition of 10% fetal calf serum. For experiments with variable glucose, DMEM medium without glucose was used and glucose was supplemented to 2 mM, 6 mM, and 16 mM values.

### Experimental treatment of cells with MOTS-c

In each experiment, the cells were incubated with varying concentrations of MOTS-c (1, 10, and 100 nM) in the experimental medium. Additionally, there was a control group where cells were not exposed to MOTS-c. The MOTS-c peptide used in the experiments was purchased from Novazym (Poznan, Poland). For the αTC-1 cells, the amino acid sequence of the mouse MOTS-c was MKWEEMGYIFL. On the other hand, for the INS-1E cells, the amino acid sequence of the rat MOTS-c was MKRKEMGYIFFSQRTLRNPL.

### Immunofluorescence

The cells underwent a series of treatments to prepare them for staining. Firstly, they were treated with 4% paraformaldehyde for 10 min and then washed three times with phosphate-buffered saline (PBS). Next, the cells were permeabilized with 1% Triton X-100 in PBS for 15 min and then washed three times with PBS. Subsequently, autofluorescence was blocked using 0.1% glycine in PBS for 15 min, and after removal, the cells were blocked with 2% bovine serum albumin (BSA) in PBS for 45 min.

For staining, the primary antibodies used were antiMOTS-c, antiMOTS-c with blocking peptide, and synthetic MOTS-c; antiinsulin (in the case of INS-1E cells); and antiglucagon (in the case of αTC1 cells). The blocking peptide (cat. no. MBS543991; MyBioSource, San Diego, CA, USA) recommended for this antiMOTS-c antibody was used, and additionally the MOTS-c peptide obtained from Novazym was added to block the MOTS-c positive signals. Antibody and blocking peptides were incubated 48 h before the use. The primary antibodies were allowed to incubate for 1 h at a concentration of 1:400 in PBS buffer with 0.2% gelatin. Following this, the cells were washed three times with PBS, and then the secondary antibody was added for 15 min also in a concentration of 1:400 in PBS buffer with 0.2% gelatin. Then, the nuclei of cells were stained with 4′,6-diamidino-2-phenylindole (DAPI) for 1 min, and photographs were taken using LSM 510 Meta, Axiovert 200 M, AxioCamHR, LD Plan Neofluar 63x/0.75 Korr Ph2 (Zeiss, Oberkochen, Germany).

The antibodies used for staining were as follows: antiinsulin, polyclonal guinea pig, cat. no. A0564 (Agilent, Santa Clara, CA, USA); antiglucagon, polyclonal guinea pig, cat. no. 4031-01F (Merck Millipore, Burlington, MA, USA); antiMOTS-c, rabbit polyclonal, cat. no. MBS542112 (MyBioSource, San Diego, CA, USA).

The following secondary antibodies were used: Alexa Fluor 488 Goat antiguinea pig IgG, cat. no. A11073 (Life Technologies, Carlsbad, CA, USA) and Cy3 Goat antirabbit IgG, cat. no. A10520 (Life Technologies, Carlsbad, CA, USA).

### MOTS-c secretion

Both types of cells were incubated in a 96-well plate and exposed to an experimental medium with the addition of (a) oleic, stearic, and palmitic acid at a concentration of 200 µM and without any fatty acid (control group), (b) 2, 6, and 16 mM of glucose. Additionally, αTC-1 cells were incubated with an experimental medium with 1, 10, and 100 nM of glucagon and the control without glucagon, and INS-1E cells were incubated with an experimental medium with 1, 10, and 100 nM of insulin, and the control group without insulin. Incubations lasted 1.5 h.

To determine the MOTS-c levels in the medium, the Rat MOTS-c kit (Sunred, Shanghai, China) and Mouse MOTS-c kit (Sunred, Shanghai, China) were measured following the instructions provided in the attached manual. To normalize the results, 3-(4,5-dimethylthiazol-2-yl)-2,5-diphenyltetrazolium bromide (MTT) assays were conducted after each incubation, and the secretion results were normalized by comparing them with the MTT results.

### Secretion of hormones

The INS-1E cells were cultured in a 96-well plate and incubated with MOTS-c for 1.5 h. Following the incubation period, the insulin levels in the medium were measured using the RI-13 K (kit from Merck Millipore, Burlington, MA, USA), according to the instructions provided in the attached manual.

Similarly, the αTC-1 cells were also cultured in a 96-well plate and incubated with MOTS-c for 1.5 h. After the incubation, the glucagon levels in the medium were measured using the GL-32 K (kit from Merck Millipore, Burlington, MA, USA), according to the instructions provided in the attached manual.

### Cell culture medium with free fatty acids

Before administering the medium to the cells, free warm fatty acids were added to the medium to achieve a concentration of 200 µM. Subsequently, the cells were incubated at 37 °C for 1.5 h to facilitate the coupling of the fatty acids to bovine serum albumin in the medium.

#### MTT

The cells were incubated for a 24-h incubation period with MOTS-c. Afterward, a 96-well plate containing the cells was used for the MTT assay. A 0.05% working solution of MTT from Merck (Darmstadt, Germany) was added to the medium in each well, and the plate was placed in an incubator for 25 min. Following the incubation, the medium was carefully removed, and the cells were dissolved in 100 µl of dimethylsulfoxide. The plate was then placed on a thermomixer at 37 °C and gently shaken for 5 min. Subsequently, the absorbance of the cells was measured at 570 nm wavelength, and background measurements were taken at 650 nm wavelength on Synergy 2 (Agilent, Santa Clara, CA, USA).

### BrdU

The cells were cultured in a 96-well plate and incubated with MOTS-c for a duration of 24 h. After this incubation period, a 5-bromo-2′-deoxyuridin (BrdU) assay was performed using the Cell Proliferation ELISA, BrdU (colorimetric) kit, according to the instructions provided in the attached manual.

### Cell death

The cells were incubated with MOTS-c for 24 h in a 96-well plate. To measure the level of cell death, a Cell Death Detection ELISA Plus test was conducted according to the instructions provided in the attached manual.

### RNA extraction

The cells were incubated with MOTS-c for 24 h in a 12-well plate. Following the incubation, RNA was extracted from the cells using Extrazol reagent purchased from BLIRT (Gdansk, Poland), and the RNA extraction protocol provided in the attached manual was followed.

### Reverse transcription

Reverse transcription was performed using the High Output cDNA Reverse Transcription Kit (Applied Biosystems, Waltham, MA, USA), as per the instructions provided in the manual.

#### PCR

The reagent used for the reaction was HOT FIREPol EvaGreen qPCR Mix Plus (Solis BioDyne OÜ, Tartu, Estonia). The reaction was carried out using the QuantStudio™ 12 K Flex System (Thermo Fisher Scientific, Waltham, MA, USA).

The primer sequences utilized (5′–3′) were: Rat insulin F: CCAGTTGGTAGAGGGAGCAG, R: AGACCATCAGCAAGCAAGCGGTC. Rat insulin receptor F: CAGAAAAACCTCTTCAGGCAAT, R: TTCAAGGGATCTTCGCTTTC. Mouse glucagon F: TACACCTGTTCGCAGCTCAG, R: TTGCACCAGCATTATAAGCAA. Mouse glucagon receptor F: GATCCGAGTACGCTCGAGGA, R: GTTGTGGTGGCATTGGTCAC. GAPDH rat F: CTGCACCACCAACTGCTTAG, R: TGATGGCATGGACTGTGG. GAPDH mouse F: ATGGTGAAGGTCGGTGTGA, R: AATCTCCACTTTGCCACTGC. The PCR reaction was conducted in a 10 µl volume, containing 5 µl of HOT FIREPol EvaGreen qPCR Mix Plus, 2 µl of the primer set (at a dilution of 2.5 µM), and 3 µl of cDNA. The PCR conditions were as follows: an initial denaturation step at 95 °C for 10 min followed by 40 cycles of denaturation at 95 °C for 15 s, annealing at 60 °C for 1 min, and extension at 72 °C for 20 s. Subsequently, a melt curve analysis was carried out with the following steps: 95 °C for 15 s, 60 °C for 60 s, and 95 °C for 15 s.

### Protein isolation

Cells were incubated with MOTS-s for 24 h on a six-well plate. Following this, they were collected using radioimmunoprecipitation assay (RIPA) buffer supplemented with protease inhibitors. The cell lysate was then vortexed and centrifuged twice and put in a thermomixer at 4 °C and shaken at 900 rpm for 10 min. After that, the lysate was centrifuged at 13,000 × *g* for 10 min. The resulting supernatant containing protein was transferred to new tubes, and protein concentration was measured with Pierce BCA Protein Assay (Thermo Fisher Scientific, Waltham, MA, USA).

### Western blot

An equal amount of protein with the addition of Laemmli and β-mercaptoethanol was separated through sodium dodecyl sulfate polyacrylamide gel electrophoresis (SDS-PAGE) 12% gel electrophoresis and then transferred onto a polyvinylidene fluoride (PVDF) Western Blotting Membrane (Roche, Basel, Switzerland). To prevent nonspecific binding, the membrane was blocked using 3% bovine serum albumin. Subsequently, the membrane was incubated overnight at 4 °C with primary antibodies (antiMOTS-c, anti-insulin receptor, and anti-glucagon receptor) at a dilution of 1:1000.

Following the primary antibody incubation, the membrane was washed with TBST buffer and then exposed to a secondary antibody (antirabbit) at a dilution of 1:5000 for 1.5 h. After another round of washing with TBST, the signal was visualized using Super Signal^™^ West Pico PLUS (Thermo Fisher Scientific, Waltham, MA, USA). Next, the membrane was incubated with an anti-β-actin antibody overnight at 4 °C, followed by washing with TBST and incubation with a secondary antibody (antimouse) for 1 h. The signal was visualized using Clarity Western ECL Substrate (Bio-Rad, Hercules, CA, USA). The visualization process was performed using the ChemiDoc MP Imaging system (Bio-Rad, Hercules, CA, USA).

The antibodies used in WB were as follows: antiMOTS-c, rabbit polyclonal, cat. no. MBS542112 (MyBioSource, San Diego, CA, USA); antiinsulin receptor middle region, rabbit polyclonal, cat. no. MBS3205807 (MyBioSource, San Diego, CA, USA); antiglucagon receptor antibody, rabbit polyclonal. cat. no. OAAF04902 (Aviva Systems Biology, San Diego, CA, USA).

The secondary antibodies used were as follows: antirabbit IgG, cat. no. 7074P2 (Cell Signaling, Danvers, MA, USA); antimouse IgG, cat. no. A2304 (Sigma Aldrich, Saint Louis, MO, USA).

## Results

### MOTS-c is present in INS-1E and αTC-1 cells

**Fig. 1:**
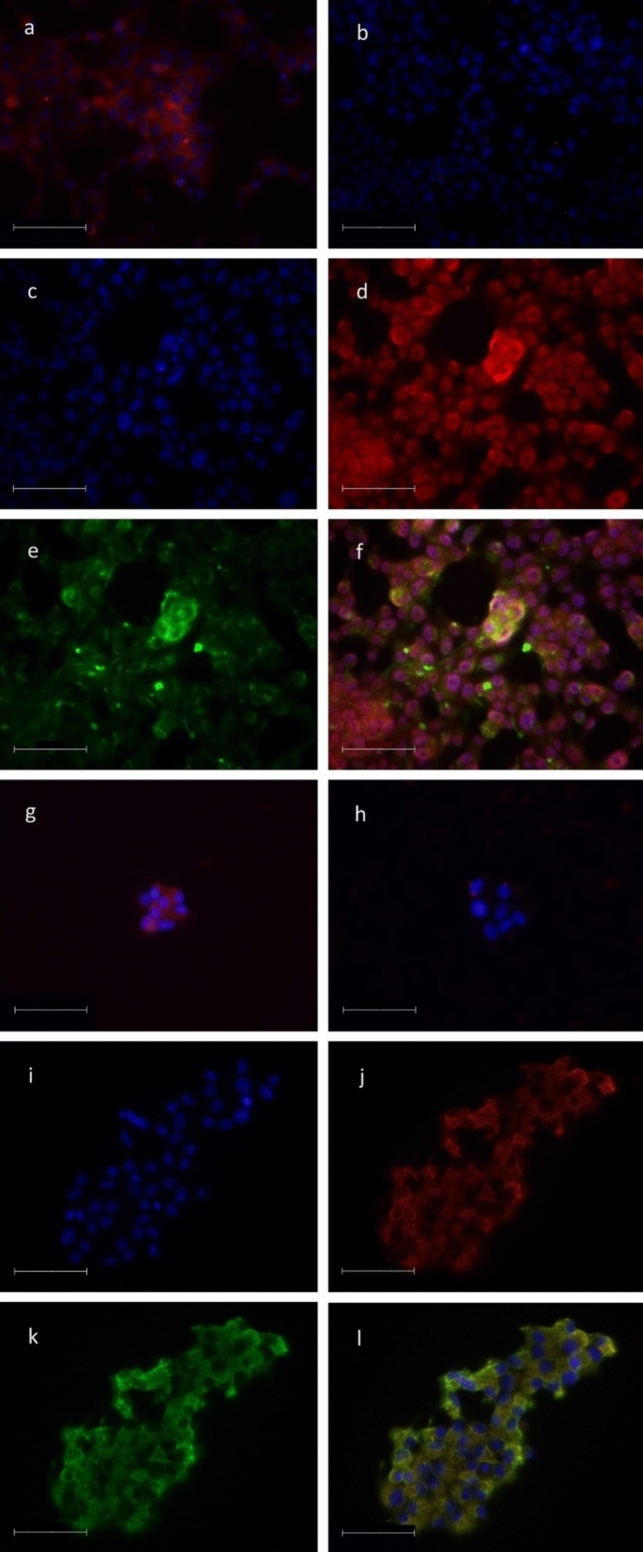
The use of antiMOTS-c antibodies demonstrates the presence of MOTS-c peptide inside the INS-1 cells (**a**), the lack of signal when blocking peptide was added (**b**), DAPI staining for INS-1E cells (**c**), the MOTS-c localization (**d**), the insulin localization (**e**), and MOTS-c colocalization with insulin (**f**). Images of MOTS-c localization (**d**) and insulin localization (**e**) were merged using ImageJ software. Analogous presence of MOTS-c was observed in αTC-1 cells (**g**), the lack of signal when blocking peptide was added (**h**), DAPI staining for αTC-1 cells (**i**), the MOTS-c localization (**j**), the glucagon localization (**k**), and MOTS-c colocalization with glucagon (**l**). Images of MOTS-c localization (**j**) and insulin localization (**k**) were merged using ImageJ software. The length of the scale bar is 50 µm

Immunofluorescence staining showed the presence of MOTS-c in both INS-1E and αTC-1 cells (Fig. 1a, g). The use of a blocking peptide abolished the specific signal from MOTS-c (Fig. 1b, h). In addition, staining was performed to indicate the presence of insulin and glucagon in INS-1 and TC-1 cells, respectively (Fig. 1e, k). The merged images from the experiment indicate the copresence of MOTS-c along with these hormones in the endocrine pancreatic cell lines (Fig. 1f, l).

### Insulin and glucagon increase MOTS-c secretion and gene expression in INS-1E and αTC-1 cells

**Fig. 2:**
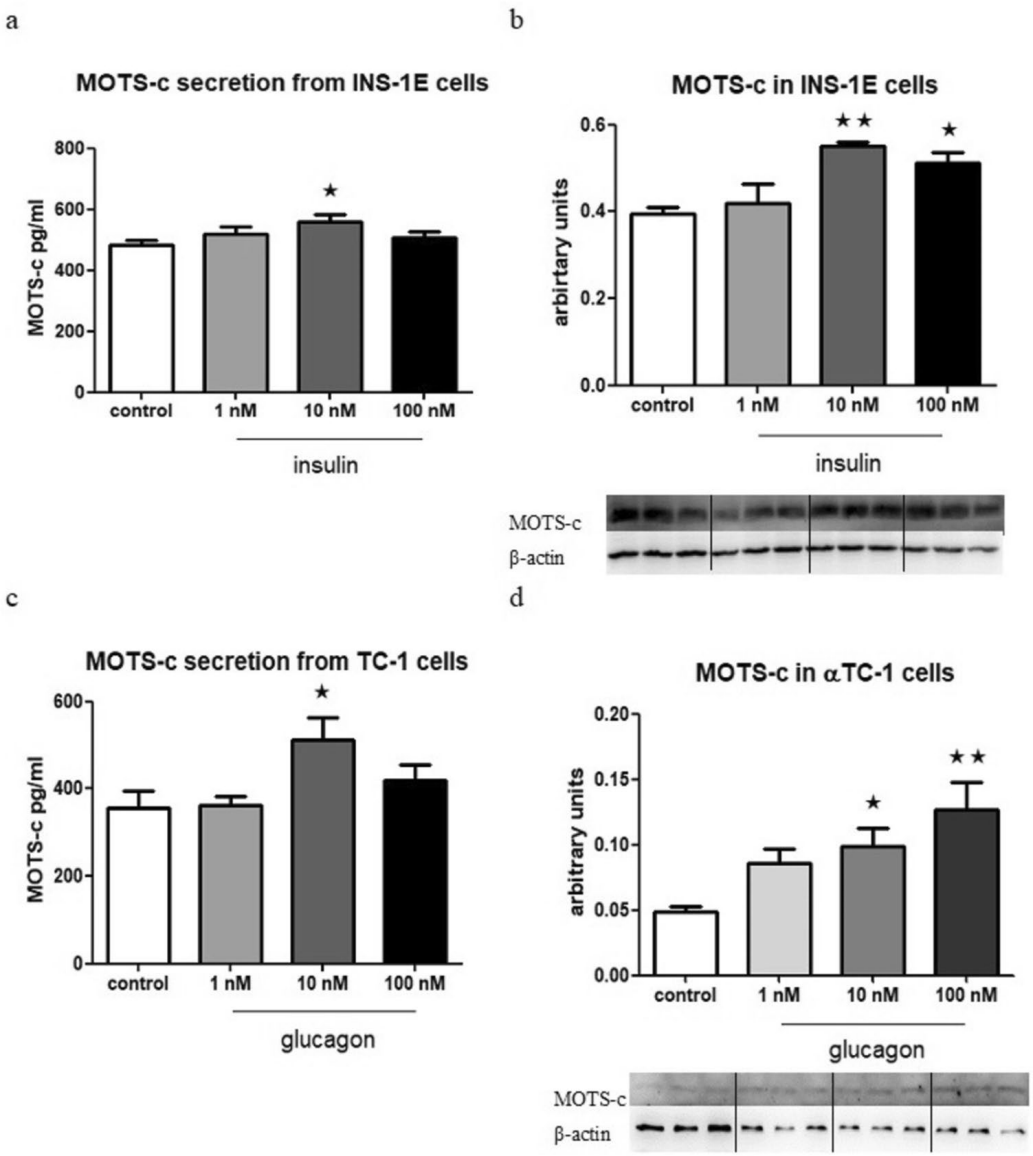
Influence of insulin on MOTS-c secretion from INS-1E cells after 1.5 h incubation, *p* = 0.0477 (**a**) and on expression at protein level in INS-1E cells after 24 h of incubation, *p* = 0.0093 (**b**). Influence of glucagon on MOTS-c secretion from αTC-1 cells after 1.5 h incubation, *p* = 0.0199 (**c**) and on the expression at protein level in αTC-1 cells after 24 h of incubation, *p* = 0.0054 (**d**). The Western blot effect presented as the ratio of the MOTS-c to the β-actin. Data show mean ± SEM, * and ** indicate *p* value < 0.05 and < 0.01, respectively

Secretion of MOTS-c was significantly increased in INS-1E cells that were incubated for 1.5 h with 10 nM insulin compared with the control group (Fig. 2a). Furthermore, the expression of MOTS-c at the protein level was significantly higher in cells that were incubated overnight with both 10 mM and 100 nM insulin, in comparison with the control group (Fig. 2b).

Similarly, in αTC-1 cells, the secretion of MOTS-c was significantly elevated after a 1.5-h incubation in a medium containing 10 nM glucagon, as compared with the control group (Fig. 2c). Moreover, the expression of MOTS-c at protein level was significantly higher in cells that were incubated overnight with both 10 mM and 100 nM glucagon, in contrast to the control group (Fig. 2d).

### MOTS-c lowers insulin gene secretion and expression and simultaneously increases insulin receptor gene expression in INS-1E cells, while MOTS-c increases glucagon gene secretion and expression in αTC-1 cells and simultaneously has no effect on glucagon receptor gene expression in αTC-1

**Fig. 3:**
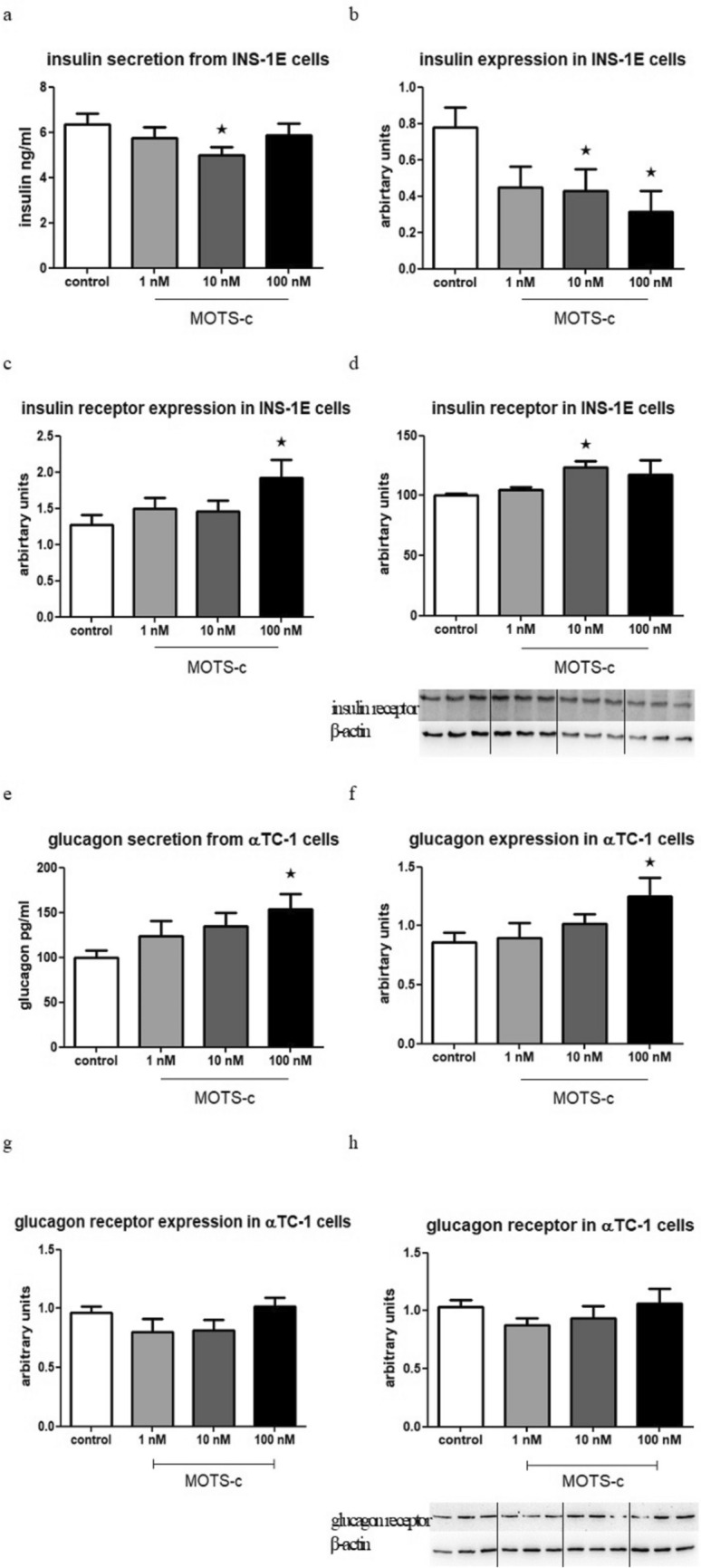
Insulin secretion from INS-1E cells after 1.5 h incubation with MOTS-c, *p* = 0.0377 (**a**), insulin gene expression in INS-1E cells after 24 h of incubation, *p* = 0.0424 (**b**), insulin receptor gene expression in INS-1E cells after 24 h of incubation with MOTS-c, mRNA level, *p* = 0.0421 (**c**), insulin receptor gene expression in INS-1E cells after 24 h of incubation with MOTS-c, protein level, *p* = 0.0491 (**d**). Glucagon secretion from αTC-1 cells after 1.5 h incubation with MOTS-c, *p* = 0.0436 (**e**), glucagon gene expression in αTC-1 cells after 24 h of incubation, *p* = 0.0417 (**f**). Glucagon receptor gene expression in αTC-1 cells after 24 h of incubation with MOTS-c, mRNA level (**g**), glucagon receptor gene expression in αTC-1 cells after 24 h of incubation with MOTS-c, protein level (**h**). The Western Blot effect presented as the ratio of the insulin or glucagon receptor to the β-actin. Data show mean ± SEM, * means *p* < 0.05

Upon treating INS-1E cells with MOTS-c for 1.5 h, a reduction in insulin secretion was observed, particularly at a concentration of 10 nM MOTS-c (Fig. 3a). Additionally, the expression of insulin mRNA was lowered in both groups treated with 10 nM and 100 nM MOTS-c (Fig. 3b). Interestingly, after overnight incubation with MOTS-c, the expression of the insulin receptor gene was enhanced, especially in the group exposed to the highest concentration of MOTS-c (Fig. 3c). However, at the protein level, the expression of the insulin receptor was higher in the group treated with 10 nM MOTS-c (Fig. 3d).

In αTC-1 cells, when treated with 100 nM MOTS-c for 1.5 h, an increase in glucagon secretion was higher than in the control group (Fig. 3e). Moreover, the expression of glucagon mRNA was increased in the group incubated with 100 nM MOTS-c after overnight incubation (Fig. 3f). Simultaneously, no effect of MOTS-c on glucagon receptor expression was observed (Fig. 3g, h).

### MOTS-c secretion and gene expression change in different glucose levels in the medium

**Fig. 4:**
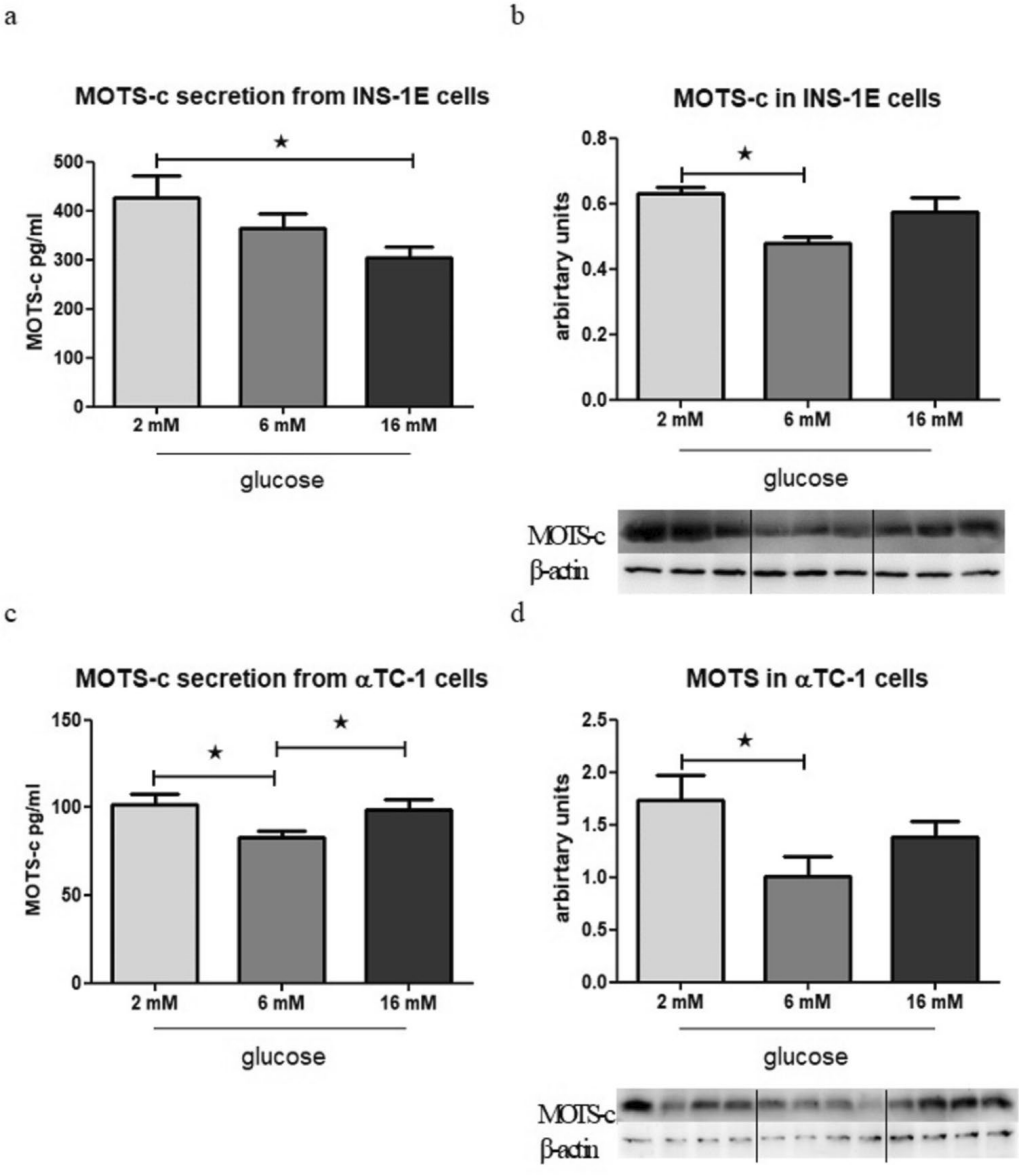
Glucose influence on MOTS-c secretion after 1.5 h from INS-1E cells, *p* = 0.0493 (**a**) and MOTS-c expression at protein level in INS-1E cells after 24 h, *p* = 0.0311 (**b**). Glucose influence on MOTS-c secretion after 1.5 h from αTC-1 cells, *p* = 0.0261 (**c**) and MOTS-c expression at protein level in αTC-1 cells after 24 h, *p* = 0.0459 (**d**). The Western Blot effect presented as the ratio of the MOTS-c to the β-actin. Data show mean ± SEM, * means *p* < 0.05

MOTS-c secretion was observed in higher amounts from INS-1E cells that were incubated for 1.5 h with 2 mM glucose when compared with the group incubated with 16 mM glucose (Fig. 4 a). Additionally, at the protein level, the expression of MOTS-c was enhanced in cells incubated with 2 mM glucose compared with the cells incubated overnight with 6 mM glucose (Fig. 4 b).

In αTC1 cells, the secretion of MOTS-c was higher when incubated with either 2 mM or 16 mM glucose than in the group incubated with 6 mM glucose (Fig. 4 c). On the protein level, the results indicate significant differences between the groups incubated with 2 mM glucose and 6 mM glucose (Fig. 4 d).

### Free fatty acids enhance MOTS-c secretion from INS-1E cells but lower it in αTC-1 cells

**Fig. 5:**
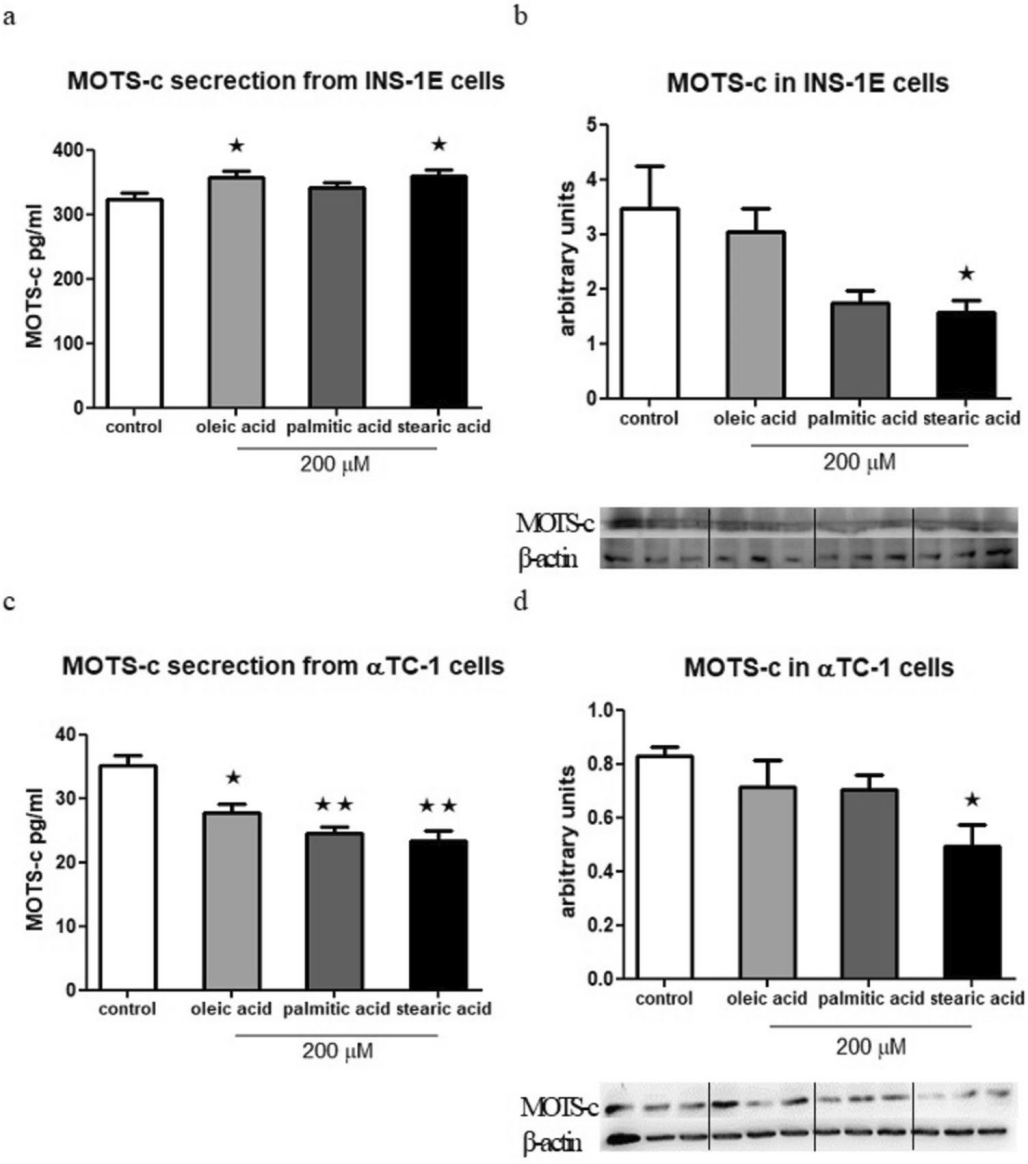
Free fatty acids influence MOTS-c secretion after 1.5 h from INS-1E cells, *p* = 0.0424 (**a**) and MOTS-c expression at protein level in INS-1E cells after 24 h,* p* = 0.0317 (**b**). Free fatty acids influence on MOTS-c secretion after 1.5 h from αTC-1 cells, *p* = 0.0015 (**c**) and MOTS-c expression at protein level in αTC-1 cells after 24 h, *p* = 0.0401 (**d**). The Western Blot effect presented as the ratio of the MOTS-c to the β-actin. Data show mean ± SEM, * and ** mean *p* < 0.05 and < 0.01, respectively

After a 1.5 h incubation with oleic and stearic acid, the secretion of MOTS-c from INS-1E cells was significantly higher (Fig. 5a). However, in the case of αTC-1 cells, the effect was the opposite, as all three examined fatty acids—oleic, palmitic, and stearic acid—result in lower MOTS-c secretion (Fig. 5c). On the other hand, after overnight incubation, in both cell lines, stearic acid appears to lower MOTS-c expression (Fig. 5b, d).

### MOTS-c enhances the viability of INS-1E cells and decreases the viability of αTC-1 cells

**Fig. 6:**
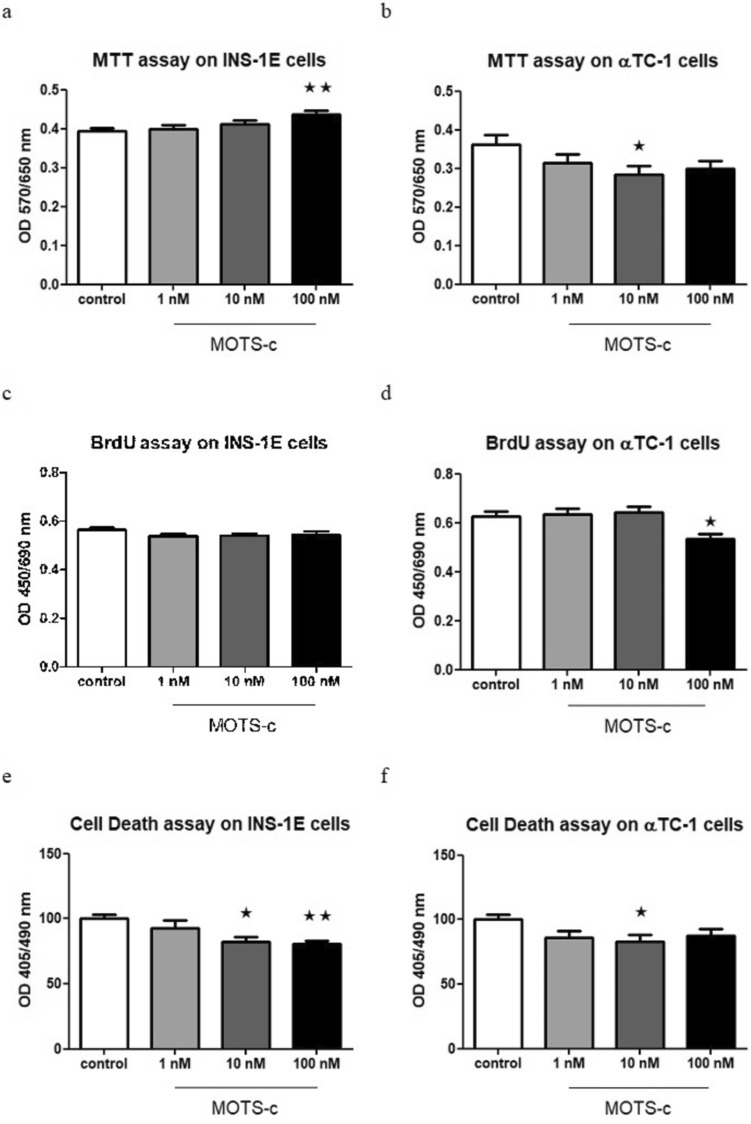
Effect of 24 h incubation with MOTS-c at doses of 1, 10, and 100 nM. MTT assay performed on INS-1E cells, *p* = 0.0096 (**a**) and αTC-1 cells, *p* = 0.0428 (**b**), BrdU assay on INS-1E cells (**c**) and αTC-1 cells, *p* = 0.015 (**d**), and cell death assay on INS-1E cells, *p* = 0.008 (**e**) and αTC-1 cells, *p* = 0.0419 (**f**). Data show mean ± SEM, * and ** mean *p* < 0.05 and < 0.01, respectively

INS-1E cells treated with 100 nM MOTS-c overnight exhibited increased viability compared with the control group (Fig. 6a). However, αTC-1 cells showed contrasting results, with reduced viability observed in groups treated with 10 nM MOTS-c after 24 h of incubation (Fig. 6b).

### MOTS-c lowers cell proliferation in αTC-1 cells but not in INS-1E

After 24 h of incubation of αTC-1 cells with MOTS-c, a decrease of cell proliferation was observed in the group treated with 100 nM peptide (Fig. 6 d). Conversely, in INS-1E cells, no significant differences in proliferation were observed (Fig. 6 c).

### MOTS-c decreases apoptosis in INS-1E and αTC-1 cells

After 24 h of incubation with MOTS-c, the results indicated that this peptide lowers apoptosis in both types of examined cells. In INS-1E cells, significant changes were observed in both the 10 and 100 nM MOTS-c groups, while in αTC-1 cells, changes were only observed in the 10 nM MOTS-c group (Fig. 6e, f).

### Supplementary data

MOTS-c alters the expression of some components of intracellular signaling pathways and transcription factors. We examined the expression of glucose transporter 2 (GluT2), cAMP response element-binding protein (CREB), and 5′-AMP-activated protein kinase (AMPK) in INS-1E cells (Supplementary Fig. 1a, c, e) and glucose transporter 1 (GluT1), Pax6 transcription factor, and MafB transcription factor in αTC-1 cells (Supplementary Fig. 1b, d, f). As our work does not describe these pathways in detail, we present them as supplementary data. Below are the primer sequences used in supplementary experiments on INS-1E cells: GluT2 F: TAGTCAGATTGCTGGCCTCAGCTT, R: TTGCCCTGACTTCCTCTTCCAACT. CREB F: GCACAGACCACTGATGGACA, R: ACGCCATAACAACTCCAGGG. AMPK F: GGTGAAGATCGGCCACTACAT, R: ATTTTCCCGACCACGTCCAG. Below are the primer sequences used in supplementary experiments on αTC-1 cells: GluT1 F: ACCCTGGGACTGCAGGTT, R: AGGGACGGAGGGCTACTG. Pax6 F: CACCAGACTCACCTGACACC, R: TCTCACACATCTGCTCACCG. Mafb F: CAGGGCTGGTTTGGAATCCT, R: TTGGCTCAATGGGAGCTCAG.

### Statistical analysis

All analyses were carried out using GraphPad Prism 6.0 software (GraphPad Software, San Diego, CA, USA). The results are presented as the arithmetic mean ± SEM. The significance of differences was determined using one-way analysis of variance (ANOVA) with a Dunnett post hoc test, comparing the results with the control group. Additionally, a Tukey post hoc test was employed when comparing groups with each other. Statistical significance is denoted by * for *p* < 0.05 and ** for *p* < 0.01.

## Discussion

MOTS-c is a novel peptide that has captured the interest of researchers since its discovery. It has been found to exert a significant impact, particularly on muscle tissue. In vitro research has demonstrated that MOTS-c enhances muscle differentiation in human (LHCN-M2) and mice (C2C12) cell lines (García-Benlloch et al. 2022). Additionally, it may act against skeletal muscle wasting induced by insulin resistance (Kumagai et al. 2021). Besides its effect on muscle tissue, MOTS-c also influences adipose tissue by promoting adipose thermogenesis to counteract hypothermia (Lu et al. 2019a). Furthermore, there are studies indicating its protective effect on pancreatic islets in type I diabetes in mice (Kong et al. 2021). Similar observations regarding the protective effect of MOTS-c on pancreatic islets and increasing insulin sensitivity in C2C12 cells were also demonstrated in the work of Yin et al. (Yin et al. 2022). At the same time, a decrease in the amount of MOTS-c peptide in the blood of people with diabetes has been observed (Ramanjaneya et al. 2019). All of these findings highlight the importance of MOTS-c as a crucial metabolic factor.

The aim of this study is to determine the influence of MOTS-c on pancreatic cell lines INS-1E and αTC-1, representing β and α cells involved in glucose metabolism. The study showed that MOTS-c is present in both INS-1E and αTC-1 cells under normal conditions (Fig. 1). This is not surprising, as MOTS-c, being a mitochondrial peptide, is also found in pancreatic endocrine cells. Although there were no specific MOTS-c-producing cells in the pancreas, it was expressed in cells producing insulin and glucagon. Considering its mitochondrial origin, it is likely that MOTS-c is distributed widely in various tissues and cells throughout the entire organism. Consequently, endogenous, as well as exogenous, peptides may impact many aspects of cellular physiology through paracrine and endocrine interactions.

This study also investigated the influence of the main pancreatic hormones on MOTS-c secretion from pancreatic cells. Both insulin and glucagon were found to enhance the secretion of MOTS-c. This suggests that MOTS-c may be cosecreted with other hormones when the secretory activity of the cell is elevated. Existing studies indicate that MOTS-c can regulate metabolism in different tissues, such as skeletal muscle (Lee et al. 2015). However, it is important to remember that INS-1E and αTC-1 cells are model cells, functioning in isolated monoculture, rather than in a more complicated environment with various types of cells, as seen in pancreatic islets, where many other hormones circulate and intercellular reactions occur. Nevertheless, the results obtained in this study demonstrate an undisturbed, direct increasing effect of pancreatic hormones on MOTS-c secretion.

The subsequent part of this research aimed to examine the effect of MOTS-c on the secretion of the two main pancreatic hormones: insulin and glucagon. The results revealed that, after incubation with MOTS-c, there was a decrease in insulin secretion and expression in INS-1E cells, while glucagon secretion and expression increased in αTC-1 cells. These findings strongly suggest that MOTS-c plays a major role in regulating pancreatic cells and glucose metabolism. Additionally, the expression of the insulin receptor was enhanced, indicating higher insulin sensitivity in β cells, which aligns with previous discoveries demonstrating that MOTS-c increases cellular insulin sensitivity (Lee et al. 2015). Additionally, the experiment showed a feedback loop between MOTS-c and insulin. Insulin increases MOTS-c secretion, while MOTS-c, in turn, lowers insulin secretion. However, no similar relationship was observed with glucagon. It is known that there are many different regulators of glucagon secretion, such as glucose, which, when released via glucagon activity, inhibits glucagon secretion (Fig. 3).

Furthermore, additional tests were conducted in this study to examine MOTS-c secretion after incubation with glucose. It is worth noting that these experiments required a change in the basal cell medium since INS-1E and αTC-1 cells are standard cultured in different glucose concentrations (11 mM and 5.6 mM, respectively). This change was necessary to examine MOTS-c secretion under hypo-, normo-, and hyperglycemic conditions, which simulate hunger, homeostasis, postprandial status, or diabetes. The results obtained from these tests on model cells demonstrated that energetic compounds can directly modulate the secretion of MOTS-c, further confirming its role in regulating metabolism.

Additionally, the study revealed that glucagon secretion increases in low glucose concentrations, and interestingly, glucagon itself acts as an enhancer of MOTS-c secretion. This finding suggests that, under conditions of hunger, characterized by low blood glucose levels and high glucagon levels, MOTS-c may serve as a stimulant for various ways of gaining energy, such as through lipolysis and fatty acids or glycogenolysis and glucose (Figs. 4 and 5).

Finally, we examined the influence of MOTS-c on the viability of pancreatic endocrine cells, which is of significant importance in type II diabetes, where beta cell damage and reduced functioning play a crucial role in disease development (Porte 1991). The results showed that MOTS-c reduces apoptosis in both types of examined cell lines, aligning with the known protective effects of MOTS-c and other MDPs (Merry et al. 2020). An interesting observation was that MOTS-c acts differently on alpha and beta cell lines concerning cell viability, as measured by the MTT assay. While it enhances viability in INS-1E cells, there is the opposite effect in αTC-1 cells. This difference could be related to the fact that MOTS-c also modulates hormone secretion in these cells, and the altered viability may be influenced by the varying levels of hormone secretion. It is plausible that cells that secrete more (glucagon from αTC-1) might exhibit lower vitality (Fig. 6).

MOTS-c has emerged as an exceptionally attractive peptide for examination. Especially its protective effect on the heart seems worth further attention (Wang et al. 2023; Zhong et al. 2022; Li et al. 2022). It ranks high on the list of potential candidates for medical use as a drug. Its unique way of affecting metabolism, leading to the nonoxidation of glucose through glycolysis, suggests that MOTS-c may have fewer disadvantages as a medicine compared with other AMPK-activating substances that could cause more side effects (Gao et al. 2023).

## Conclusions

The direct impact of MOTS-c on pancreatic cell functioning has not been examined previously, but MOTS-c is a very significant regulator of pancreatic function. The results obtained from this study provide good fundamentals for further experiments. While cell lines serve as common models for initial experiments, the next step should involve experiments with isolated pancreatic islets to observe how MOTS-c influences pancreatic physiology in a more complex and physiological environment beyond cell culture conditions.

### Supplementary Information

Below is the link to the electronic supplementary material.

Supplementary file1 Supp. Fig. 1. Effect of 24 h incubation with MOTS-c in doses 1, 10, and 100 nM on Glut2, CREB, and AMPK expression in INS-1E cells (Sup. Fig. 1a, c, e, *p* value = 0.0451, 0.0455 and 0.0482, respectively) and GluT1, Pax6 and Mafb in αTC-1 cells (Sup. Fig. 1b, d, f, *p* value = 0.0436, 0.0475 and 0.0465, respectively). Data show mean ± SEM, * means *p* <0.05. (TIF 605 KB)

## Data Availability

The data that support the findings of this study are not openly available and are available from the corresponding author upon reasonable request.
