# Supplementary figures and images for: MOTS-c regulates pancreatic alpha and beta cell functions in vitro
Source: Histochem Cell Biol. 2024 Mar 2;161(6):449–60. doi: 10.1007/s00418-024-02274-0 (PMC11162381; doi:10.1007/s00418-024-02274-0)

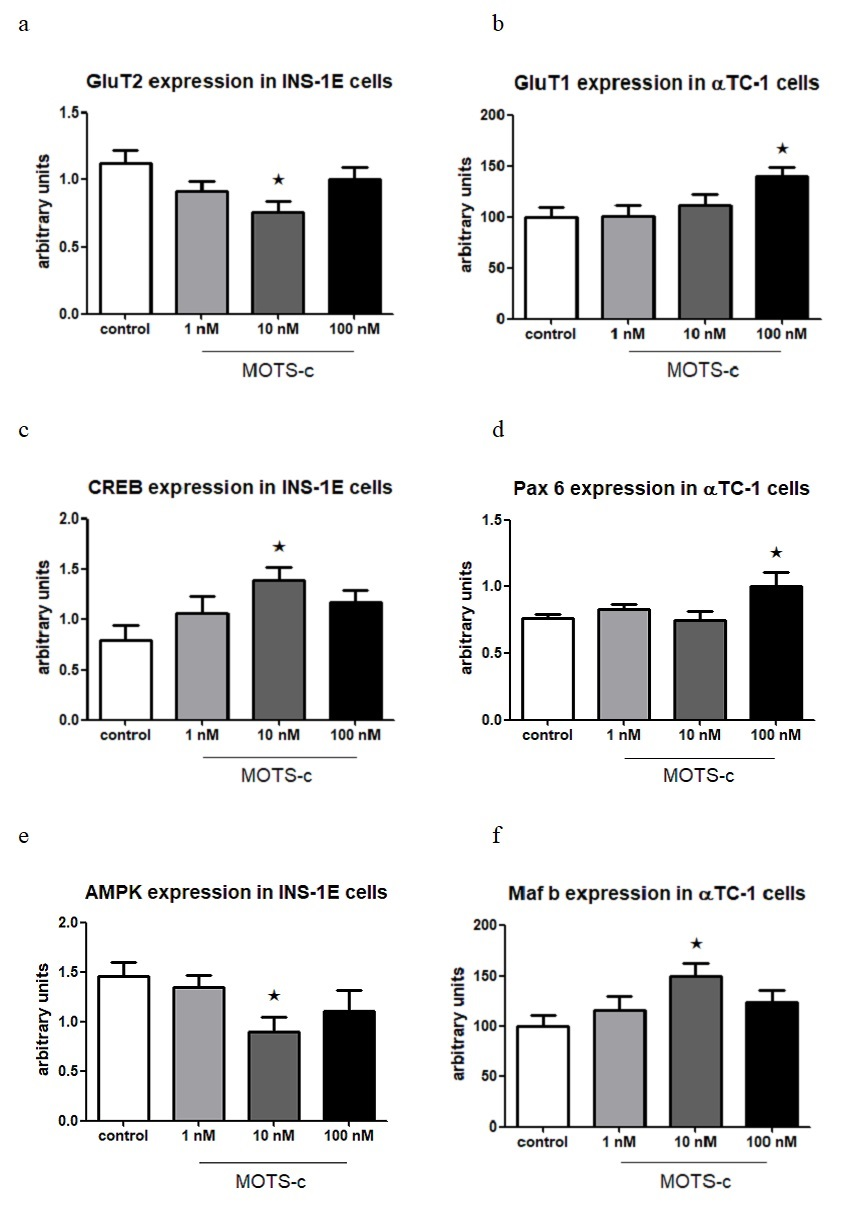

Supplement: Supplementary file 1 — Supplementary file1 Supp. Fig. 1. Effect of 24 h incubation with MOTS-c in doses 1, 10, and 100 nM on Glut2, CREB, and AMPK expression in INS-1E cells (Sup. Fig. 1a, c, e, p value = 0.0451, 0.0455 and 0.0482, respectively) and GluT1, Pax6 and Mafb in αTC-1 cells (Sup. Fig. 1b, d, f, p value = 0.0436, 0.0475 and 0.0465, respectively). Data show mean ± SEM, * means p <0.05. (TIF 605 KB) [file 418_2024_2274_MOESM1_ESM.tif]
